# Supplementary material for: Causal Relationship between Aging and Anorexia Nervosa: A White-Matter-Microstructure-Mediated Mendelian Randomization Analysis
Source: Biomedicines. 2024 Aug 16;12(8):1874. doi: 10.3390/biomedicines12081874 (PMC11351342; doi:10.3390/biomedicines12081874)
Supplement: Supplementary file 1 [file biomedicines-12-01874-s001.zip › Supplementary Figure/Figure S4/Singe SNP plot of MR analysis for TL to ubm-b-1512.pdf]

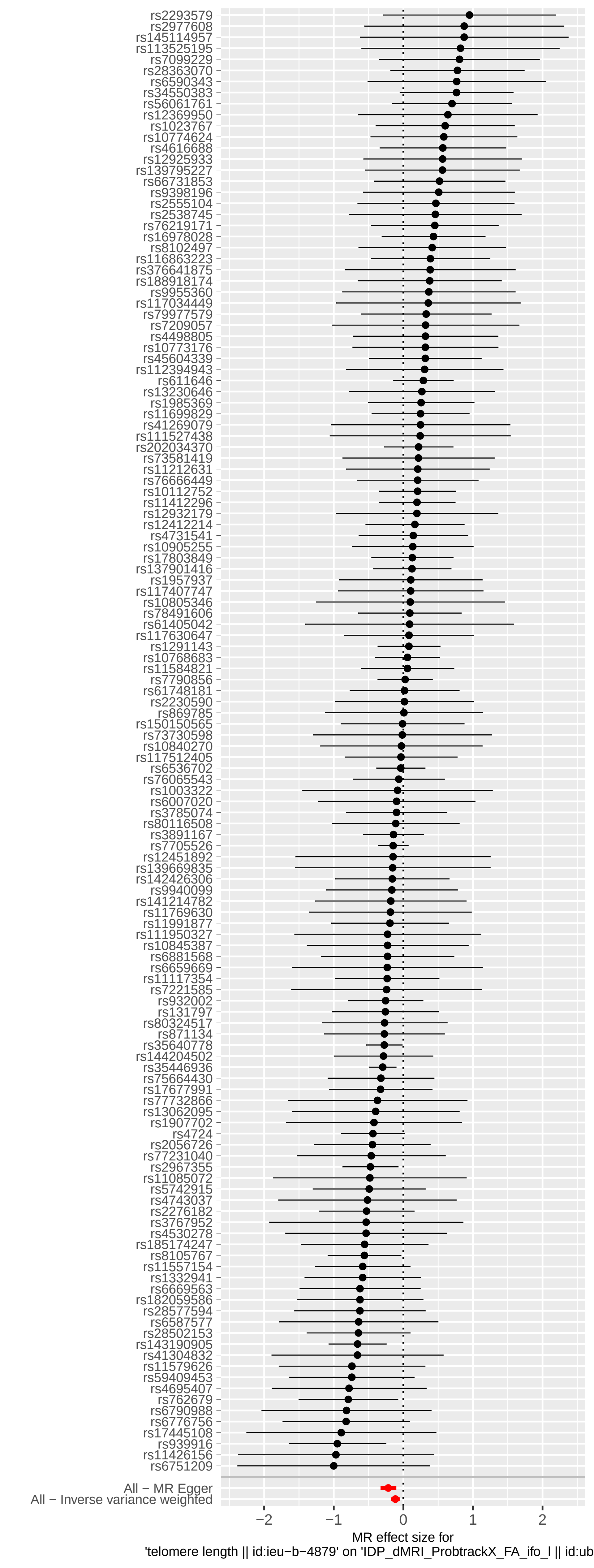
rs2293579
rs2977608
rs145114957
rs113525195
rs7099229
rs28363070
rs6590343
rs34550383
rs56061761
rs12369950
rs1023767
rs10774624
rs4616688
rs12925933
rs139795227
rs66731853
rs9398196
rs2555104
rs2538745
rs76219171
rs16978028
rs8102497
rs116863223
rs376641875
rs188918174
rs9955360
rs117034449
rs79977579
rs7209057
rs4498805
rs10773176
rs45604339
rs112394943
rs611646
rs13230646
rs1985369
rs11699829
rs41269079
rs111527438
rs202034370
rs73581419
rs11212631
rs76666449
rs10112752
rs11412296
rs12932179
rs12412214
rs4731541
rs10905255
rs17803849
rs137901416
rs1957937
rs117407747
rs10805346
rs78491606
rs61405042
rs117630647
rs1291143
rs10768683
rs11584821
rs7790856
rs61748181
rs2230590
rs869785
rs150150565
rs73730598
rs10840270
rs117512405
rs6536702
rs76065543
rs1003322
rs6007020
rs3785074
rs80116508
rs3891167
rs7705526
rs12451892
rs139669835
rs142426306
rs9940099
rs141214782
rs11769630
rs11991877
rs111950327
rs10845387
rs6881568
rs6659669
rs11117354
rs7221585
rs932002
rs131797
rs80324517
rs871134
rs35640778
rs144204502
rs35446936
rs75664430
rs17677991
rs77732866
rs13062095
rs1907702
rs4724
rs2056726
rs77231040
rs2967355
rs11085072
rs5742915
rs4743037
rs2276182
rs3767952
rs4530278
rs185174247
rs8105767
rs11557154
rs1332941
rs6669563
rs182059586
rs28577594
rs6587577
rs28502153
rs143190905
rs41304832
rs11579626
rs59409453
rs4695407
rs762679
rs6790988
rs6776756
rs17445108
rs939916
rs11426156
rs6751209
All − MR Egger
All − Inverse variance weighted
−2
−1
0
1
2
MR effect size for
'telomere length || id:ieu−b−4879' on 'IDP_dMRI_ProbtrackX_FA_ifo_l || id:ub
